# Flow Cytometry as a Rapid and Valuable Method in Investigation of Colistin Resistance in Carbapenem-Resistant *Klebsiella pneumoniae* Isolates

**DOI:** 10.3390/antibiotics13050418

**Published:** 2024-05-02

**Authors:** Şafak Ceren Uçak, Betigül Öngen

**Affiliations:** 1 Medical Microbiology Department, İstanbul Faculty of Medicine, İstanbul University, 34093 İstanbul, Türkiye; ongenb@istanbul.edu.tr; 2 Medical Microbiology Department, Faculty of Medicine, İstanbul Nişantaşı University, 34398 İstanbul, Türkiye

**Keywords:** colistin, DiBAC_4_(3), flow cytometry, *Klebsiella pneumoniae*, MIC

## Abstract

Rapid detection of antimicrobial resistance is crucial for early initiation of appropriate therapy. The aim of this study was to investigate whether resistance to colistin, the last-resort antibiotic, in carbapenem-resistant *Klebsiella pneumoniae* (CRKP) isolates can be detected accurately and rapidly by flow cytometry (FCM). The VITEK 2 automated system was used to identify 85 *K. pneumoniae* strains and to determine their resistance to carbapenems. The minimum inhibitory concentration (MIC) values for colistin in 85 CRKP strains were determined by broth microdilution (BMD), which is the reference method. In addition, FCM was used, combined with DiBAC_4_(3) fluorescent stain, to determine colistin susceptibility. The MIC₅₀ value of the strains, 80% of which were resistant to colistin by the BMD method, was 16 mg/L, and the MIC₉₀ value was 32 mg/L. When FCM was compared with the reference method, it was determined that the specificity was 94.1%, sensitivity was 100% of FCM, and Cohen’s kappa value was 0.96. Colistin susceptibility results with FCM were obtained within an average of 2 h. These findings suggest that FCM holds great promise as a rapid and reliable alternative method for detecting colistin resistance in CRKP strains.

## 1. Introduction

Over the last several decades, the emergence of resistance in microorganisms has accelerated due to the widespread use and misuse of antibacterial drugs [1]. The development of resistance supported by empirical treatment has complicated treatment, prolonged length of hospital stays, raised mortality rates, and increased costs. Many bacteria, which are *Enterobacteriaceae* members producing extended-spectrum beta-lactamase (ESBL), have also become resistant to other antimicrobial agents. As a result of the increasing use of carbapenems for bacteria that develop multidrug resistance, carbapenem resistance appears to be on the rise worldwide [2]. The World Health Organization has declared *K. pneumoniae* resistant to third-generation cephalosporins and carbapenems as one of the nine bacteria of concern due to its resistance to antibacterial drugs commonly used in treatment and has grouped resistant bacteria in the critical priority class in the list of global priority pathogens for which new treatments are urgently needed [1,3].

In these circumstances, polymyxins (colistin) have become a treatment option and even an antibiotic of last resort for multidrug-resistant *K. pneumoniae* infections in clinics. With the increasing use of colistin, the treatment of *K. pneumoniae* infections has become a global problem [4,5]. Disk diffusion and gradient test methods, which are commonly used in laboratories, fail to detect colistin-resistant organisms due to poor diffusion of colistin into the agar medium [6]. The Antibiotic Susceptibility Committees (Clinical and Laboratory Standards Institute (CLSI) and European Committee on Antimicrobial Susceptibility Testing (EUCAST)) recommend broth microdilution (BMD) for antimicrobial susceptibility testing (AST) of colistin [7,8,9]. Nevertheless, the time-consuming nature of this method (18–24 h), its lack of convenience for routine use, and its inability to yield reliable results from automated systems have increased the necessity for methods that yield accurate results in a shorter period of time in routine laboratories. This is crucial for the prevention of empirical treatment errors and resistance development [10].

Flow cytometry (FCM) is widely used in immunology laboratories. Currently, the use of this technique is expanding to identify bacteria individually and provide rapid information on the integrity and viability of cell particles and antibiotic-treated bacteria [11,12]. FCM has previously been used to study antimicrobial susceptibility [12,13,14], but its widespread use for AST is only recently being adopted [15,16,17,18]. Recent technological developments and studies show that FCM can be used to assess cellular viability and membrane potential in bacterial populations [11]. Research on rapid and accurate detection of antibiotic-resistant bacteria, as well as to reduce the spread of resistance, based on FCM analyses has increased considerably in recent years [17,18,19]. Susceptibility studies conducted with antibiotics other than colistin using this method, which has been introduced as an alternative method, have indicated that results can be achieved in as short as 2 to 4 h [13,20,21]. 

The aim of the present study was to investigate whether colistin resistance in carbapenem-resistant *Klebsiella pneumoniae* (CRKP) strains can be determined accurately and rapidly by FCM and whether this method is convenient for routine use in clinical microbiology laboratories in order to ensure that antibiotic treatment is initiated in a shorter time and with the appropriate antibiotic.

## 2. Results

### 2.1. Antibiotic Susceptibility Results for K. pneumoniae Strains

Eighty-five isolates identified as *K. pneumoniae* by the VITEK 2 system were carbapenem-resistant (ertapenem > 4). All carbapenem-resistant *K. pneumoniae* strains were resistant to cefazolin, cefuroxime, ceftazidime, and piperacillin/tazobactam, 98.8% to ciprofloxacin, 88.2% to amikacin, 87% to trimethoprim-sulfamethoxazole, and 83.5% to gentamicin.

### 2.2. Colistin MIC Results Determined by the Broth Microdilution Method

The minimum inhibitory concentration (MIC) values determined for colistin in 85 carbapenem-resistant *K. pneumoniae* strains were evaluated by the BMD according to EUCAST criteria, and 68 (80%) of the strains were resistant. The present study showed that the MIC values for colistin in positive and negative control strains were 4 mg/L for *E. coli* NCTC 13846 (*mcr*-1+) and 0.5 mg/L for *P. aeruginosa* ATCC 27853, respectively. Table 1 shows the MIC distributions and MIC₅₀–MIC₉₀ values of susceptible (S) and resistant (R) isolates. 

### 2.3. Colistin Susceptibility Results Determined by the Flow Cytometry Method

**Figure 1:**
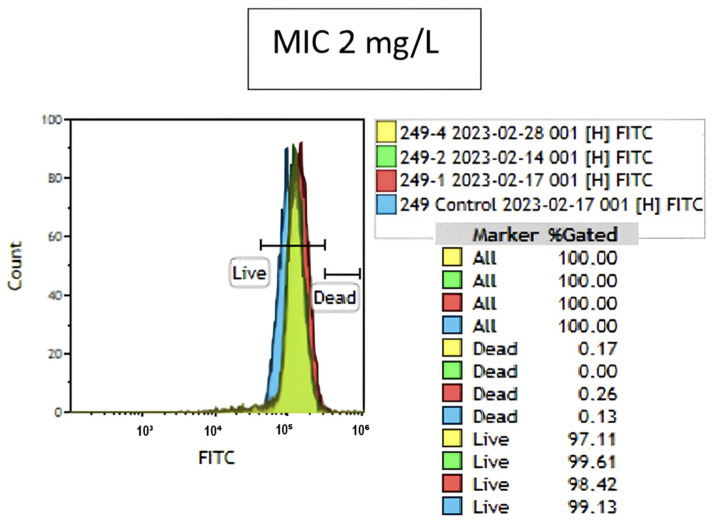
Overlay graph of the isolate with a major error (blue indicates results for the growth control tube; red, 1 mg/L; green, 2 mg/L; and yellow, 4 mg/L colistin containing tube).

The colistin susceptibility results of FCM revealed that 69 of 85 *K. pneumoniae* isolates were resistant and 16 were susceptible (see Supplementary Materials). When compared with the BMD method, a major error was detected in one of the isolates by FCM (Figure 1). Even though the MIC value of the isolate found by the BMD method was 2 mg/L (S), it was found to be resistant (R) by FCM. The strain with a major error was analyzed twice, and the same result was obtained. 

**Figure 2:**
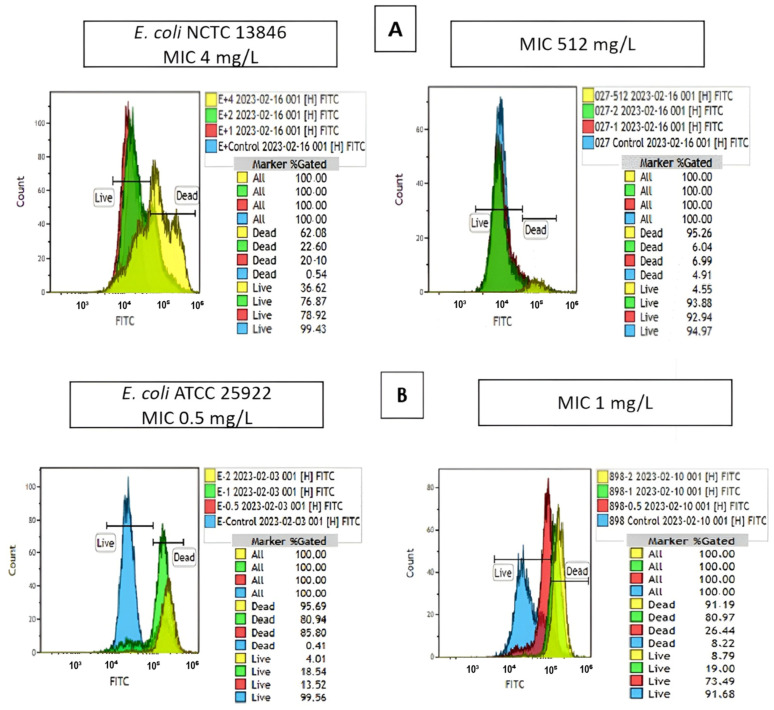
Overlay graphs of the reference strains (*E. coli* NCTC 13846 (*mcr*-1+), *E. coli* ATCC 25922) and the clinical strains with MIC values of 512 mg/L (R) and 1 mg/L (S). (**A**) indicates the results for resistant strains; blue indicates the growth control tube; red, 1 mg/L; green, 2 mg/L; and yellow, 4 mg/L indicate the tube containing colistin. (**B**) indicates the results for sensitive strains; blue indicates the growth control tube; red, 0.5 mg/L; green, 1 mg/L; and yellow, 2 mg/L indicate the tube containing colistin.

Figure 2 shows one of the overlay graphs of the reference strains and the overlay graphs of the clinical strains with MIC values of 512 mg/L (R) and 1 mg/L (S).

The McNemar (χ^2^) test cross-table shows the colistin resistance results determined by FCM and BMD methods in 85 *K. pneumoniae* isolates in the present study (Table 2).

When FCM was compared with the reference method, it was determined that the sensitivity was 100%, specificity was 94.1%, positive predictive value was 98.5%, negative predictive value was 100%, very major error was 0%, and major error was 5.8% of FCM; also, Cohen’s kappa value was 0.96. In studies with a confidence interval ≥ 95%, a very large error < 3% and a large error < 7% are considered statistically significant [22]. The Cohen’s kappa analysis, with a concordance force between 0.81 and 1.00, indicates almost perfect agreement [23]. In the Cohen’s kappa test analysis, we found almost perfect categorical agreement between BMD, which is the reference method for determining antibiotic resistance, and FCM, which we used in this study (κ = 0.96).

## 3. Discussion

Most of the ESBL-producing *Enterobacteriaceae* are resistant to third-generation cephalosporins as well as other antimicrobial agents such as aminoglycosides, trimethoprim, and quinolones. The increasing use of carbapenems for such multidrug-resistant bacteria has gradually led to an increase in carbapenem resistance [2]. As a result of increasing carbapenem resistance, the clinical use of polymyxins (colistin and polymyxin B) has increased, which have become antimicrobial agents of last resort to treat multidrug-resistant infections, often as a part of combination therapies [8,24,25]. 

Due to that polymyxins are large cationic molecules and diffuse poorly into agar, the problem of the inability to accurately determine susceptibility in diffusion-based assays frequently used in routine laboratories has been raised [6,25], and incompatible results have begun to be reported in various studies using both disk diffusion and gradient test methods in comparison with the reference method [26,27,28]. Therefore, disk diffusion or gradient test (E-test, etc.) methods cannot be used for the detection of colistin resistance in *K. pneumoniae* (and other *Enterobacterales* and nonfermentive bacteria such as *Pseudomonas* and *Acinetobacter*). Although automated systems such as VITEK 2, Phoenix, MicroScan, etc., can be easily used in routine laboratories, the reliability of these systems in the detection of colistin resistance remains controversial [26,27,29,30].

Recommended by CLSI and EUCAST to detect colistin susceptibility, the currently recognized reference method is the BMD [8,31]. Although it is important to provide data on colistin MIC value for treatment decisions when alternative antimicrobial agents are unavailable due to resistance, the time-consuming nature of these recommended methods restricts their routine use [32,33]. 

As an alternative method, FCM has been reported to yield results that are compatible with the reference method when compared with other susceptibility methods, and it has also been shown that results can be achieved in a short time, such as 2–4 h in general, in antimicrobial susceptibility studies using FCM [13,20,21,34,35,36].

FCM was previously used to study antimicrobial susceptibility [12,13,20], but with recent technological developments (dye variety, software, etc.), its use has become widespread [15,16,17,18,35].

In a study conducted by e Silva et al. [35] with FCM, which they defined as an “ultra-rapid AST”, they examined colistin susceptibility in 116 Gram-negative (*Enterobacterales* (12 *E. coli*, 38 *K. pneumoniae*, 21 *Enterobacter* spp., 3 *Proteus* spp., 2 *Morganella morganii*, 1 *Providentia rettgeri* and 1 *Serratia marcescens*), 28 *P. aeruginosa*, and 10 *A. baumannii*) bacteria and reference strains and compared the results with the BMD method. The researchers reported that the two methods yielded compatible results with each other and emphasized that the results were reached in 16–24 h with the BMD, and this time was reduced to 1.5 h with FCM.

In another study with FCM, the colistin susceptibilities of 174 strains (53 *E. coli*, 57 *K. pneumoniae*, 34 *P. aeruginosa*, and 30 *Acinetobacter* spp.) were initially determined by BMD, and then the cells were stained with YoPro-1 fluorescent dye and were evaluated by FCM. The categorical agreement was found to be very good. The researchers reported that FCM results were 75% more rapid than BMD [15]. On the other hand, they assessed the antibiotic susceptibilities of clinical *E. coli* and *Staphylococcus epidermidis* isolates and two reference strains by FCM using the DiBAC_4_(3) stain. The results of this study, in which the tube titration (macrodilution) method was used as a reference method, showed that antibiotic-induced membrane potential damage of bacteria can be demonstrated within 2–5 h, depending on the species, and it is possible to assess antibiotic susceptibility by FCM [13].

Inglis et al. [17] studied FCM and SYTO9. They examined amikacin, aztreonam, ciprofloxacin, colistin, cefepime, gentamicin, imipenem, levofloxacin, meropenem, piperacillin-tazobactam, trimethoprim-sulfamethoxazole, ceftazidime, and tobramycin susceptibility in 27 Gram-negative, 15 Gram-positive bacteria, and reference strains (*E. coli*, *K. pneumoniae*, *S. aureus*) and compared the results with the BMD method. The researchers reported a categorical agreement of 91% and an essential agreement of 100%. 

Suller et al. [20] used FCM and DiBAC_4_(3) stains to determine the susceptibility of five methicillin-resistant *S. aureus* (MRSA) strains and two susceptible reference strains to penicillin G, methicillin, and vancomycin. The results of the study showed that after being treated with vancomycin, all MRSA isolates exhibited increased fluorescence, whereby it was considered that MRSA was susceptible to vancomycin. The results obtained with the FCM method were found to be compatible with the BMD.

The reason why a DiBAC_4_(3) fluorescent stain was used in the present study is the ability of this stain to detect bacterial membrane potential changes caused by antibiotic treatment. DiBAC_4_(3), a lipophilic anion, has a low binding capacity for intact membranes and only penetrates the cell by binding to lipid-rich intracellular components when the membranes are depolarized so that the cells become more and more fluorescent [13]. As a result of the present study, it was reported that live and dead cells could be clearly differentiated by using DiBAC_4_(3) for colistin; we determined only one major error.

In the present study, the BMD, the reference method, was used to determine the MIC values. Several studies from Türkiye that used the BMD method to determine the MIC values of colistin in *Enterobacterales* members are available in the literature [37,38,39]. Yis [37] reported that 48.18% of 110 carbapenem-resistant *K. pneumoniae* strains were resistant to colistin, according to BMD results. On the other hand, the colistin resistance rate in the present study was found to be 80%, which was higher than other studies. This may probably be due to some of our isolates being recovered from patients during the COVID-19 pandemic, the period in which the antibiotic was used intensively.

There are only few antimicrobial susceptibility studies using FCM in Türkiye [16,34,40]. In these studies, automated Phoenix, gradient tests (E-test), and FCM were used to determine the susceptibility of 174 clinical isolates of *K. pneumoniae* (87 carbapenem-resistant and 87 carbapenem-susceptible) to meropenem [40]. For the FCM study, TO (Thiazole orange), which stains live and dead cells together, and PI (Propidium iodide), which stains only live cells, were selected as stains. As a result of the study, it was reported that live and dead cells could be clearly differentiated by using TO and PI stains together; the agreement between FCM and the E-test was very good; very major error was found in only one isolate. Another study investigated the antibiotic susceptibilities of 11 clinical isolates and 6 reference strains by FCM using SYTO 9 and PI stain [16]. The results of this study, in which BMD was used to determine the MIC values, showed compatible data between BMD and FCM except for two major errors. It was also underlined that FCM would shorten the time and the results could be achieved on the same day. 

Apart from the current study, no other research has been found from Türkiye that specifically examined colistin susceptibility using FCM and compared the results with the reference method. There are a few studies in the world in which susceptibility to colistin was investigated with FCM [15,17,35,41]. Therefore, the present study is important for the validation of the FCM method. As emphasized in these studies, the time required to obtain results is significantly shorter compared to the reference method. It took approximately 2 h to determine colistin susceptibility with FCM in the present study. Also, the Cohen’s kappa test analysis result showed that FCM can be used precisely and reliably in routine microbiology laboratories.

## 4. Materials and Methods

### 4.1. Sample Size and Selection of Strains

Similar studies were taken into consideration to determine the number of strains, and the acceptable sampling error was set at d = 0.15. Eighty-five carbapenem-resistant *K. pneumoniae* strains were included in the study as a result of the calculation made at power of 80% and confidence interval of 95% using G*Power version 3.1.9.7. For the study, approval was obtained from the Ethics Committee of Istanbul Medical Faculty, Istanbul University, with decision no. E-29624016-050.99-477801, dated 20 September 2021. 

The study was conducted with *K. pneumoniae* strains, which were isolated from clinical patient samples sent to the bacteriology laboratory of the Department of Medical Microbiology, İstanbul Medical Faculty, for a routine examination and determined to be resistant to carbapenems. Only one isolate from each patient was included in the study.

### 4.2. Identification by the VITEK 2 Automated System and Determination of Carbapenem Susceptibility

In the present study, the VITEK 2 (BioMérieux, Marcy l’Etoile, France) automated system was used for identification of the bacteria at the species level and determination of resistance to antibiotics other than colistin (Cefazolin, cefuroxime, ceftazidime, piperacillin/tazobactam, ciprofloxacin, amikacin, trimethoprim-sulfamethoxazole, and gentamicin). The 85 *K. pneumoniae* strains were categorized as susceptible and resistant according to the MIC breakpoints for carbapenems [9].

### 4.3. Determination of Minimum Inhibitory Concentration (MIC) Values for Colistin by Broth Microdilution (BMD)

The BMD was performed according to CLSI and EUCAST standards to define the MIC that inhibits visible bacterial growth [7,31]. Standard powder of colistin sulfate (Biosynth-Carbosynth, Staad, Switzerland, AC20542; potency: 23.576 IU/mg) was dissolved in sterilized distilled water, and stock solution was prepared and stored at −80 °C until use. Polystyrene 96-well plates (Laborant, İstanbul, Türkiye) were prepared with twofold serial dilutions of colistin in cation-adjusted Mueller Hinton broth (MHB) (Becton Dickinson, New York, NY, USA), to obtain a concentration range of 64–0.125 mg/L. The bacterial suspension from fresh cultures at a turbidity of 0.5 McFarland (1 × 10^8^ CFU/mL) was then inoculated into each well to achieve a final concentration of 5 × 10^6^ CFU/mL. The reference control strains, *Escherichia coli* NCTC 13846 (*mcr*-1+) and *Pseudomonas aeruginosa* ATCC 27853, were included in each plate. The inoculated microplates were incubated at 35 °C ± 2 °C for 16–20 h in an ambient air incubator. Testing was repeated for isolates with skipping phenomenon was observed. The results were interpreted according to the EUCAST clinical cut-off values, and isolates with MIC ≤ 2 mg/L and MIC > 2 mg/L were considered susceptible and resistant, respectively [9,31]. 

### 4.4. Optimization Studies for FCM

**Figure 3:**
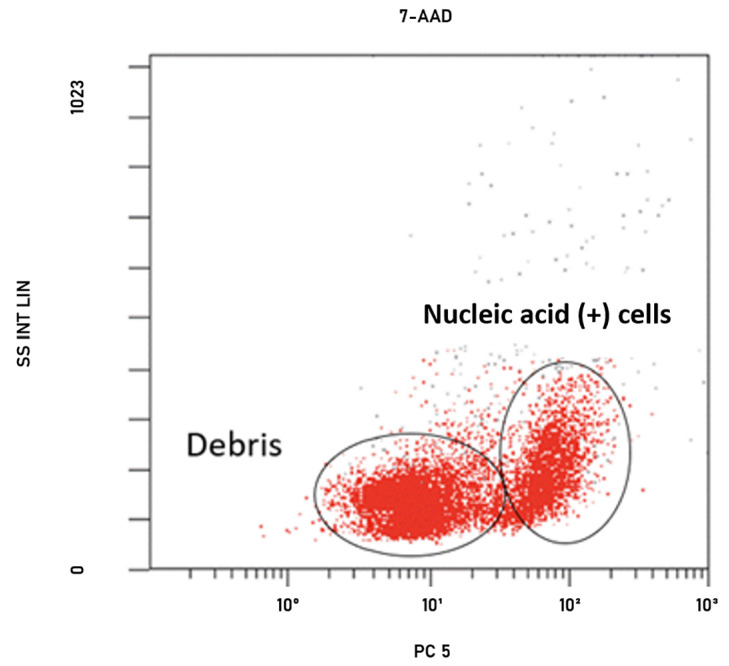
Dot plot graph of the gating process of cells stained with fluorescent dye (7-AAD) in the SSC/PC5 channel of *K. pneumoniae* strain.

Firstly, the autofluorescence caused by the natural structure of the cells was checked to see whether it was at the level that would affect the study. For this, antibiotic-free and unstained bacterial suspensions were analyzed with FCM in the fluorescein isothiocyanate (FITC) channel. After the analysis, no autofluorescence was noted to affect flow cytometry studies. After these preliminary studies, the bacteria were stained with 7-amino actinomycin D (7-AAD) (BD Biosciences Pharmingen, San Diego, CA, USA) stain without the addition of antibiotics to differentiate debris or nucleic acid-positive cells, and the results were analyzed in the PC5 channel. The 7-AAD fluorescence of nucleic acid positive cells were measured on FL-3 (Figure 3) [42]. After analysis, the space to be gated was detected in the dot plot graph. 

The inoculum concentration recommended for the BMD was used as the bacterial concentration in the study [7,31]. For each isolate, the first tube (growth control tube) stained with DiBAC_4_(3) (Cayman Chemical Company, Ann Arbor, MI, USA) stain, to which no antibiotic was added, was read on the flow cytometry. The first gating was made on the dot plot graph, and necessary arrangements were made on the device to ensure that the same area was always gated in the results of the dot plot of the other tubes to which different amounts of antibiotics were added.

### 4.5. Determination of Colistin Susceptibility by FCM

The NAVIOS EX (Beckman Coulter, Brea, CA, USA) device was used to detect colistin resistance by FCM. Four FCM tubes were used to test different antibiotic concentrations for each strain. After preparing the turbidity of bacterial suspensions from fresh cultures equivalent to a 0.5 McFarland standard in cation-adjusted MHB, the bacterial suspensions were diluted 1:100 to ensure that the final bacterial concentrations were 1 × 10^6^ CFU/mL in the four tubes used for each strain [11,34]. 

The first tubes prepared for all strains that were determined to be resistant to BMD (MIC > 2 mg/L) were determined to be growth control tube. Briefly, 500 μL of antibiotic were added to the second, third, and fourth tubes, with concentrations of 1 mg/L, 2 mg/L, and MIC values, respectively. The first tubes prepared for the strains found to be susceptible to BMD (MIC ≤ 2 mg/L) were determined as the growth control tube, and 500 μL of antibiotic at concentrations of 0.5 mg/L, 1 mg/L, and 2 mg/L were added to the second, third, and fourth tubes, respectively. Tubes were incubated at 35 °C ± 1 for 30 min. After the incubation, 2 μL of DiBAC_4_(3) (5 mg/L final concentration) stain was added to all tubes, and the samples were kept in the dark for 15 min at room temperature to stain the bacteria [13,21]. Positive and negative control tubes containing reference strains (*E. coli* NCTC 13846 (*mcr*-1+), *E. coli* ATCC 25922) were prepared for each study. At the end of the staining process, each vortexed tube was read in the flow cytometry device using DiBAC_4_(3), and fluorescence of viable cells was measured on FL-1 (488 nm wavelength laser with the FITC filter block). The maximum emission wavelength of DiBAC_4_(3) is 516 nm and was measured by the green fluorescence detector. For each isolate, firstly, the growth control tube was read, and gating was carried out on the dot plot graph. Also, quadrants were placed on the histogram graph of the growth control tube to indicate the live and dead cell zones, and the percentage of these cells in the total cell population was determined. The same quadrants were always used in the histogram results of other tubes with different antibiotic dilutions. The histogram plots of the four tubes with different antibiotic dilutions for each isolate were converted into overlay graphs, and the results were evaluated on these plots. Beckman Coulter-Kaluza 2.2 analysis software was used to convert histogram plots into overlay graphs.

Since no standardized breakpoint is available for the determination of antibiotic susceptibility by FCM, colistin-sensitive isolates were evaluated as dead cells with impaired cell membrane potential and stained with DiBAC_4_(3) stain, and resistant isolates were evaluated as live cells with intact cell membrane potential according to the reference method, BMD. Susceptibility evaluations were based on whether the strains had similar (susceptible (dead)) or different (resistant (live)) histogram plot patterns with the growth control tubes, and the plots generated for the reference strains were evaluated first in each study. According to the results of the preliminary experimental studies, the breakpoint between live and dead cells was determined as 50%.

### 4.6. Statistical Analysis

The rate confirmed positive for FCM should not be statistically different from the BMD rate, which is the reference method. The McNemar (χ^2^) test was used to compare paired proportions. Cohen’s kappa test was used to find the categorical agreement between BMD, which is the reference method, and the FCM we used in the study. Statistical analysis was performed with SPSS v.26 statistical software.

## 5. Conclusions

The shorter AST time is critical for the rapid detection of resistant bacteria and the early initiation of treatment, which in turn contributes to a drop in morbidity and mortality rates. In addition, it is clear that there would be benefits such as a shorter length of hospital stay, a reduction in patient care costs, and a lower workload for physicians and laboratory staff. FCM, which has proven specificity and sensitivity in many fields, has disadvantages such as not being available in every laboratory, incomplete standardization for AST and the need for experienced personnel. FCM is already used in immunology laboratories, but is not widely used in clinical microbiology laboratories as it has not yet been validated and standardized. On the other hand, based on the data we have gathered by comparing it with the reference method, we believe that the validation of this method will be completed upon further studies focusing on different clinical strains, different fluorescent stains, and different antimicrobials, and with the support of appropriate computer software programs, it is likely to be placed into routine use in the near future. 

## Figures and Tables

**Table 1:** Colistin MIC distributions of carbapenem-resistant *K. pneumoniae* isolates detected by the broth microdilution method.

	Susceptible							Resistant						MIC₅₀–MIC₉₀	
**Colistin MIC (mg/L)**	0.03	0.06	0.125	0.25	0.5	1	2	4	8	16	32	128	512	16	32
**Number (n)**	1	2	2	2	3	2	5	5	12	29	20	1	1		
**Total**	17 (20%)							68 (80%)							

**Table 2:** McNemar (χ^2^) test cross-tabulation.

Broth Microdilution (BMD)			
**Flow Cytometry (FCM)**	R^1^	S^1^	Total
R^2^	68	1	69
S^2^	0	16	16
Total	68	17	85

R^1^: resistant at BMD; S^1^: susceptible at BMD; R^2^: resistant at FCM; S^2^: susceptible at FMC.

## Data Availability

The data are included in the article by excluding personal data that may not comply with GDPR regulations.

## Supplementary Materials

The following supporting information can be downloaded at: https://www.mdpi.com/article/10.3390/antibiotics13050418/s1, Table S1: Colistin MIC values of 85 CRKP isolates detected by the BMD and comparison of sensitivity results of FCM and BMD.
