# Supplementary material for: Flow Cytometry as a Rapid and Valuable Method in Investigation of Colistin Resistance in Carbapenem-Resistant Klebsiella pneumoniae Isolates
Source: Antibiotics (Basel). 2024 May 2;13(5):418. doi: 10.3390/antibiotics13050418 (PMC11117237; doi:10.3390/antibiotics13050418)
Supplement: Supplementary file 1 [file antibiotics-13-00418-s001.zip › antibiotics-2972743-supplementary.pdf]

Supplemental Table S1. Colistin MIC values of 85 CRKP isolates detected by the BMD and comparison of sensitivity results of FCM and BMD.

Isolates Number (n=85)	Colistin			
	SMD		FCM	Comment
	MIC (mg/L)	R/S	R/S	
E. coli NCTC 13846	4	R	R	CA
E. coli ATCC 25922	0.5	S	S	CA
027	512	R	R	CA
762	128	R	R	CA
292	32	R	R	CA
763	32	R	R	CA
926	32	R	R	CA
284	32	R	R	CA
559	32	R	R	CA
545	32	R	R	CA
241	32	R	R	CA
570	32	R	R	CA
989	32	R	R	CA
710	32	R	R	CA
502	32	R	R	CA
936	32	R	R	CA
088	32	R	R	CA
278	32	R	R	CA
688	32	R	R	CA
725	32	R	R	CA
766	32	R	R	CA
785	32	R	R	CA
511	32	R	R	CA
826	32	R	R	CA
741	16	R	R	CA
163	16	R	R	CA
291	16	R	R	CA
054	16	R	R	CA
322	16	R	R	CA
7985	16	R	R	CA
805	16	R	R	CA
849	16	R	R	CA
8725	16	R	R	CA
714	16	R	R	CA
280	16	R	R	CA
986	16	R	R	CA
880	16	R	R	CA
928	16	R	R	CA
854	16	R	R	CA
104	16	R	R	CA
318	16	R	R	CA

100	16	R	R	CA
249	16	R	R	CA
071	16	R	R	CA
789	16	R	R	CA
897	16	R	R	CA
159	16	R	R	CA
985	16	R	R	CA
844	16	R	R	CA
867	16	R	R	CA
848	16	R	R	CA
405	16	R	R	CA
585	16	R	R	CA
665	8	R	R	CA
409	8	R	R	CA
852	8	R	R	CA
394	8	R	R	CA
057	8	R	R	CA
403	8	R	R	CA
825	8	R	R	CA
0725	8	R	R	CA
963	8	R	R	CA
940	8	R	R	CA
439	8	R	R	CA
384	8	R	R	CA
429	4	R	R	CA
512	4	R	R	CA
866	4	R	R	CA
550	4	R	R	CA
878	4	R	R	CA
**249**	**2**	**S**	**R**	**ME**
505	2	S	S	CA
734	2	S	S	CA
493	2	S	S	CA
611	2	S	S	CA
898	1	S	S	CA
777	1	S	S	CA
356	0.5	S	S	CA
959	0.5	S	S	CA
765	0.5	S	S	CA
022	0.25	S	S	CA
221	0.25	S	S	CA
078	0.125	S	S	CA
745	0.125	S	S	CA
153	0.0625	S	S	CA
841	0.0625	S	S	CA
289	0.03125	S	S	CA

S, susceptible; R, resistant; CA, categorical agreement, ME, major error
